# Arctic deep-sea hydrothermal microbiomes as a natural niche for novel antimicrobial peptides

**DOI:** 10.1186/s12866-026-05098-1

**Published:** 2026-05-08

**Authors:** Thuc Trong Nguyen, Ida Helene Steen, Maren Helene Bøe, Marit Otterlei, Runar Stokke

**Affiliations:** 1 Department of Biological Sciences, Centre for Deep-Sea Research, University of Bergen, Bergen, Norway; 2 Department of Clinical and Molecular Medicine, Norwegian University of Science and Technology, Trondheim, Norway

**Keywords:** Extreme microbiomes, Arctic Mid-Ocean Ridges, Antimicrobial peptides, Metagenomics, Metatranscriptomics, Deep-sea hydrothermal vents, Antimicrobial resistance

## Abstract

**Background:**

The escalating threat of antimicrobial resistance (AMR) has created an urgent need for new antimicrobial agents. Antimicrobial peptides (AMPs) are promising alternatives to conventional antibiotics due to their broad-spectrum activity and reduced risk of resistance development. While most AMP discovery efforts have focused on terrestrial microbes, extreme environments remain largely untapped. Deep-sea hydrothermal vent biofilms, such as those from the Arctic Mid-Ocean Ridges (AMOR), are unique ecosystems characterized by high pressure, temperature gradients, and chemical extremes. These conditions select for microorganisms with specialized adaptations, including the production of bioactive compounds that confer survival advantages. Such peptides may exhibit enhanced stability and novel mechanisms of action, making hydrothermal biofilms an exceptional resource for next-generation antimicrobials.

**Results:**

Using metagenomic and metatranscriptomic datasets from nine recently published AMOR biofilms, we predicted 961 AMP sequences with Macrel, of which 873 were unique and showed no identity to entries in the Antimicrobial Peptide Database (APD). AMPs were distributed across 51 microbial phyla, including underrepresented archaeal groups such as *Asgardarchaeota*, *Nanoarchaeota*, and *Micrarchaeota*. Transcriptomic profiling detected AMP expression in 25 phyla, including low-abundance candidate taxa, highlighting active AMP production. In silico minimum inhibitory concentration (MIC) prediction using APEX 1.1 suggested that 16.7% of AMPs may inhibit at least one clinically relevant pathogen, with *Acinetobacter baumannii* emerging as the most susceptible. Four peptides were synthesized for experimental validation; AMP OLKFNNDA_52_10 exhibited moderate in vitro activity against *Staphylococcus aureus* and weak activity against *Escherichia coli*, while showing low cytotoxicity toward human HEK293 cells. Other tested peptides displayed weak or no activity, underscoring discrepancies between computational predictions and biological outcomes.

**Conclusions:**

Our study reveals extensive taxonomic and structural diversity of AMPs in Arctic hydrothermal vent biofilms and identifies novel candidates withbioactive potential. These findings emphasize the importance of integrating metagenomics, transcriptomics, machine learning, and experimental validation to uncover bioactive compounds from underexplored microbial ecosystems. Overall, AMOR biofilms represent a rich and untapped source of AMPs, offering new opportunities for antimicrobial drug discovery in the fight against AMR.

**Supplementary Information:**

The online version contains supplementary material available at 10.1186/s12866-026-05098-1.

## Background

In Russian and Finnish folklore, a live frog was traditionally placed in a container of milk to prevent it from souring. Centuries later, scientists discovered that this practice had a biochemical basis: the frog’s skin secretes AMPs that inhibit the growth of fermentative microbes [1]. Since then, AMPs have been explored across multiple fields, as alternative antibiotics in medicine, as food preservatives in the food industry, and as crop protectants in agriculture [2, 3]. AMPs are short, typically composed of 10–50 amino acids, naturally occurring peptides that provide innate immune defence across microorganisms, plants, fungi, and animals [4, 5]. Based on their structures, AMPs can be categorised into five classes: linear extended, α-helical, β-structured, mixed α/β-structured, and complex topologies (e.g., cyclic peptides, lasso peptides, etc.) [2, 3]. AMPs exhibit broad-spectrum antimicrobial activity, targeting diverse pathogens. AMPs inhibit pathogens through various mechanisms, such as membrane disruption, DNA binding, interference with protein synthesis, enzyme inhibition, cell wall synthesis inhibition, and immunomodulatory effects [2, 3]. AMPs have gained increasing attention due to their broad-spectrum antimicrobial activities, including antibiotic (e.g., daptomycin, nisin) [6, 7], antiviral (e.g., protegrin, indolicidin) [2], antifungal (e.g., pea defensin Psd1, magainin-2) [8, 9], and anticancer properties (e.g., ecteinascidin 743, α-Defensin-1) [10, 11]. AMPs are considered alternatives to conventional antibiotics. Their diverse targets make it difficult for pathogens to develop specific resistance mechanisms, and their biodegradability makes them more environmentally friendly by reducing pharmaceutical pollution [3, 12].

The development of antibiotics revolutionized medicine, but the rapid emergence of antimicrobial resistance (AMR) has become a major global threat, especially as the discovery of new drug classes has dwindled [13]. This growing resistance poses a significant challenge to the future of medical practice worldwide [13, 14]. With the help of modern technologies, scientists are now searching for new antibiotics in diverse environments, from garden soil to extreme habitats, and have discovered several antimicrobial compounds from understudied microbial taxa [15–18]. Especially, recent studies suggest that archaea represent a largely untapped reservoir for antimicrobial discovery, with multiple AMPs from this domain demonstrating in vitro activity [15, 18]. Recently, we conducted a systematic study on the biosynthetic gene clusters (BGCs) of microbes inhabiting nine biofilms from Arctic deep-sea hydrothermal vents located along the AMOR [19]. Our results revealed that microbial BGCs from this extreme environment are particularly rich in ribosomally synthesized and post-translationally modified peptides (RiPP) and nonribosomal peptides (NRP) classes, which are known sources of AMPs [6, 7, 16, 20]. Building on these findings, we performed an ad hoc search for AMPs in the corresponding biofilm samples and compared them with experimentally validated AMPs from public databases.

Using Macrel (Metagenomic AMP Classification and Retrieval) [21], we identified 961 AMPs from MAGs associated with AMOR biofilms, including 873 unique sequences. AMPs were identified in 51 microbial phyla, including 9 archaeal and 42 bacterial phyla. None of the AMPs from AMOR biofilms matched reference AMPs in the Antimicrobial Peptide Database (ADP) [22] when clustered using CD-HIT [23], suggesting they have distinct structures. Nanopore sequencing of cDNA revealed that many AMPs predicted by Macrel were transcriptionally expressed. AMPs were detected for the first time in the archaeal phylum *Asgardarchaeota*, supported by transcriptional expression data. One AMP from our dataset showed a promising minimum inhibitory concentration (MIC) against pathogenic bacteria, while exhibiting low cytotoxicity toward human cells. Our results highlight the richness of archaea as a previously overlooked source of AMPs and present AMOR biofilms as a valuable bioresource for the discovery of new antibiotics. Further testing is needed on the diverse AMP structures identified in our study to assess their potential in combating AMR.

## Methods

### Metagenomic and metatranscriptomic data collection

The metagenomic and metatranscriptomic datasets analyzed in this study were previously generated as part of a study on the biosynthetic potential of Arctic deep-sea hydrothermal biofilms [19]. Briefly, samples were collected from three hydrothermal vent locations: Seven Sisters (1 sample), Loki’s Castle (1 sample), and Aegir vent field (7 samples). The full dataset of all the MAGs used in this study, as well as their affiliated taxonomic information, is presented in Supplementary Table 1. DNA and RNA were extracted and sequenced using a combination of Illumina and Nanopore sequencing platforms and subjected to quality control, metagenome-assembled genome (MAG) reconstruction, and taxonomic classification using GTDB version 2.5.2 with taxonomic database R226, as described in [19]. In this study, we build upon this dataset by specifically investigating AMPs within these biofilms.

### Antimicrobial peptide prediction

AMP prediction was performed using Macrel v1.2.0 on AMOR biofilm MAGs with the following command “contigs --log-append --keep-fasta-headers” [21]. Macrel extracted small open reading frames (smORFs) from contigs using its modified version of Prodigal. AMP candidates were then predicted using a machine-learning model trained on physicochemical and compositional features. Only peptides with a prediction probability above 50% were retained. The resulting AMPs were assigned to their respective MAGs for taxonomic classification. Contigs that were not incorporated into MAGs were also analysed for AMP prediction using Macrel and were labelled as ‘exMAGs’. Redundant sequences were clustered, and redundancy was removed using CD-HIT v4.8.1 [23] with a 95% similarity threshold (parameters: -c 0.95 -n 5), and only non-redundant sequences were retained for further analysis.

### Novelty of AMPs derived from AMOR biofilms

To assess the novelty of the predicted AMPs identified from MAGs, all sequences were compared against experimentally validated AMPs in the 2024 release of the APD [22]. This comparison was conducted using CD-HIT with clustering parameters -c 1 -n 5, ensuring 100% sequence identity.

To evaluate sequence similarity between predicted and known AMPs, we constructed a pairwise similarity matrix based on global alignment scores using the *PairwiseAligner* module from Biopython (v1.85), which implements the Needleman–Wunsch algorithm for global alignment. The similarity matrix was normalized using the *MinMaxScaler* function from Scikit-learn (v1.5.2) to ensure consistency and enhance the performance of downstream visualizations. Dimensionality reduction was performed using Uniform Manifold Approximation and Projection (UMAP) [24] with the parameters n_neighbors = 20, min_dist = 0.1, spread = 10.0, and metric=’precomputed’, reducing the high-dimensional similarity data to two dimensions. This approach effectively preserves both local and global relationships in the data, enabling intuitive visualization of peptide similarity. We used the 2024 release of APD, which contains 3,306 non-redundant, experimentally validated peptide sequences, as a reference for known AMPs. Five FDA-approved AMPs, Nisin, Gramicidin, Daptomycin, Defensin, and Penetratin, were highlighted to benchmark the predicted peptides derived from AMOR biofilms. To enhance the interpretability of the plot, kernel density estimation (KDE) contours were applied to highlight the distribution densities of APD3 and inMAG peptides. The final UMAP plot was generated using Matplotlib (v3.10.1) and Seaborn (v0.13.2), with data processing performed in Python (v3.13) using NumPy (v2.2.4) and pandas (v2.2.3).

### AMP expression profiling

AMP expression profiling was performed using modified Macrel scripts. All smORFs corresponding to predicted AMPs were first extracted from the Macrel output. These smORFs were then mapped to their respective metatranscriptomes using Minimap2 v2.17-release 941 with parameters *-a -x map-ont*. A new *longreads-abundance* module was added to the Macrel pipeline by modifying the main.py script, in accordance with Macrel’s license. The modified script is in the online repository provided in the Data Availability section. Read per kilobase (RPK) and transcripts per million (TPM) values were subsequently calculated based on the mapping results. This analysis utilized Nanopore cDNA long reads from the seven available biofilm samples as previously described [19].

### Prediction of MIC values for antibacterial activity

The potential antibacterial activity of peptides was assessed using the Antibiotic Peptide de-Extinction (APEX) deep learning framework, version 1.1 [15, 25]. APEX 1.1 is a regression-based deep learning model trained on experimentally validated MIC data from peptides tested against a panel of clinically relevant bacterial strains [15], primarily members of the ESKAPE group [26], due to their clinical significance. The training panel included *Acinetobacter baumannii* ATCC 19,606, *Escherichia coli* ATCC 11,775, AIC221, and AIC222, *Klebsiella pneumoniae* ATCC 13,883, *Pseudomonas aeruginosa* PA01 and PA14, *Staphylococcus aureus* ATCC 12,600 and methicillin-resistant *S. aureus* (MRSA) ATCC BAA-1556, as well as vancomycin-resistant *Enterococcus faecalis* ATCC 700,802 *and E. faecium* ATCC 700,221 [15]. To estimate the antimicrobial potency of AMOR AMPs, APEX 1.1 was run under default settings to predict MIC values in silico. The predicted MIC values were used to prioritize peptides with potentially high antimicrobial activity for further investigation.

### Selection and synthesis of AMPs for validation

To evaluate the antibacterial activity of AMPs predicted by Macrel, we randomly selected four peptides from our dataset of 961 sequences, using the following criteria. All selected peptides had Macrel-predicted AMP probabilities ranging from moderate to high confidence. Two peptides were predicted to possess antibacterial activity by APEX 1.1: one with high predicted potency against multiple pathogens, and one with predicted activity against a single bacterial species. The remaining two peptides were predicted by APEX 1.1 to have no antibacterial activity (mean MIC > 100 µM). This selection strategy aimed to assess the performance and predictive accuracy of both machine learning tools (i.e., Macrel and APEX 1.1) by comparing in silico predictions with in vitro experimental outcomes. The four selected peptide sequences were synthesised by Innovagen AB (Sweden).

### MIC determination

MICs were determined following the standard protocol for antimicrobial susceptibility testing as established by the Clinical and Laboratory Standards Institute (CLSI) [27], with minor modifications. Briefly, bacterial suspensions were prepared in cation-adjusted Mueller-Hinton Broth (CAMHB; St. Olavs Hospital), adjusted to 0.5 McFarland, and further diluted to 5 × 10⁵ CFU/mL. Peptides were 2-fold serially diluted and added to polypropylene 96-well microtiter plates (Greiner) at 10% of the total well volume, followed by the addition of 100 µL of the bacterial suspension. Plates were incubated at 37 °C for 24 h, and bacterial growth was assessed by visual inspection. Two independent biological replicates were performed.

### Viability assay

The growth inhibiting effect of the peptides on transformed human embryonic kidney epithelial (HEK293 – ATCC-CRL-1573 – American Type Culture Collection) cells, was tested using the PrestoBlue™ viability assay (Invitrogen) following the guidelines provided by the manufacturer. Briefly, 3000 cells/well were seeded in 96-well plates and incubated (37 °C, 5% CO2, humidified atmosphere) for a minimum of 4 h to adhere. The cells were either treated with peptide (6 µM, 8 µM, 10 µM) or left untreated as a control before further incubation. IC50, i.e., the peptide dose that gave 50% growth compared to untreated control, was measured by fluorescence (excitation at 544 nm, emission at 590 nm) after 72 h.

### Statistical analysis

Statistical comparisons of AMP distributions between inMAGs and exMAGs were performed using the Wilcoxon signed-rank test for paired sample comparisons, implemented in SciPy (v1.15.2). Differences in peptide class abundance between CLP and CDP in the inMAGs dataset were also assessed using the same test. A significance threshold of *p* < 0.05 was used.

To assess differences in AMP expression levels across metatranscriptomic samples, we performed nonparametric statistical testing. Expression values in TPM were log-transformed before analysis. A Kruskal–Wallis test was used to determine whether peptide expression distributions differed significantly among samples. Where significance was detected, post hoc Dunn’s tests with Holm–Bonferroni correction for multiple comparisons were conducted using the *scikit-posthocs* Python package (v0.11.4).

### Data availability

All data generated and analysed during this study are included in this published article (and its supplementary information files). The datasets analysed in the current study was recently published [19] and are available via BioProjects; PRJNA1301805, PRJNA881934, PRJNA1301797, PRJNA1301837, PRJNA1301809, PRJNA1301812, PRJNA1301820, PRJNA1301824 and PRJNA1301830, with MAG sequence data and raw sequencing data deposited at NCBI. The modified main.py script for longread-abundance, along with all source code and data used for statistical analyses, is available on GitHub (https://github.com/trongthucnguyen/DeepSeaQuence_biofilms ).

## Results

In a recent study, we discovered a diverse set of BGCs from the AMOR biofilms, many of which belong to product groups known to encode AMPs. From that study, we obtained 1,140 non-redundant MAGs that passed quality thresholds, including 124 archaeal and 1,016 bacterial MAGs [19]. In summary, the dataset showed a rich microbial diversity, comprising 51 bacterial phyla and 10 archaeal phyla. *Pseudomonadota* was the most abundant bacterial phylum, followed by *Bacteroidota*, *Campylobacterota*, *Aquificota*, *Desulfobacterota*, *Chloroflexota*, and *Planctomycetota*. Although archaeal phyla were less diverse and abundant than bacterial counterparts, they were notable for the diversity of *Thermoproteota*, followed by other extremophile-associated phyla such as *Halobacteriota*, *Asgardarchaeota*, and *Nanobdellota*. The diversity of the AMOR biofilm microbiota, coupled with the richness of its biosynthetic potential [19], provides a strong foundation for investigating its capacity to produce antimicrobial compounds, specifically AMPs.

### AMPs from Arctic hydrothermal vent biofilms

**Fig. 1:**
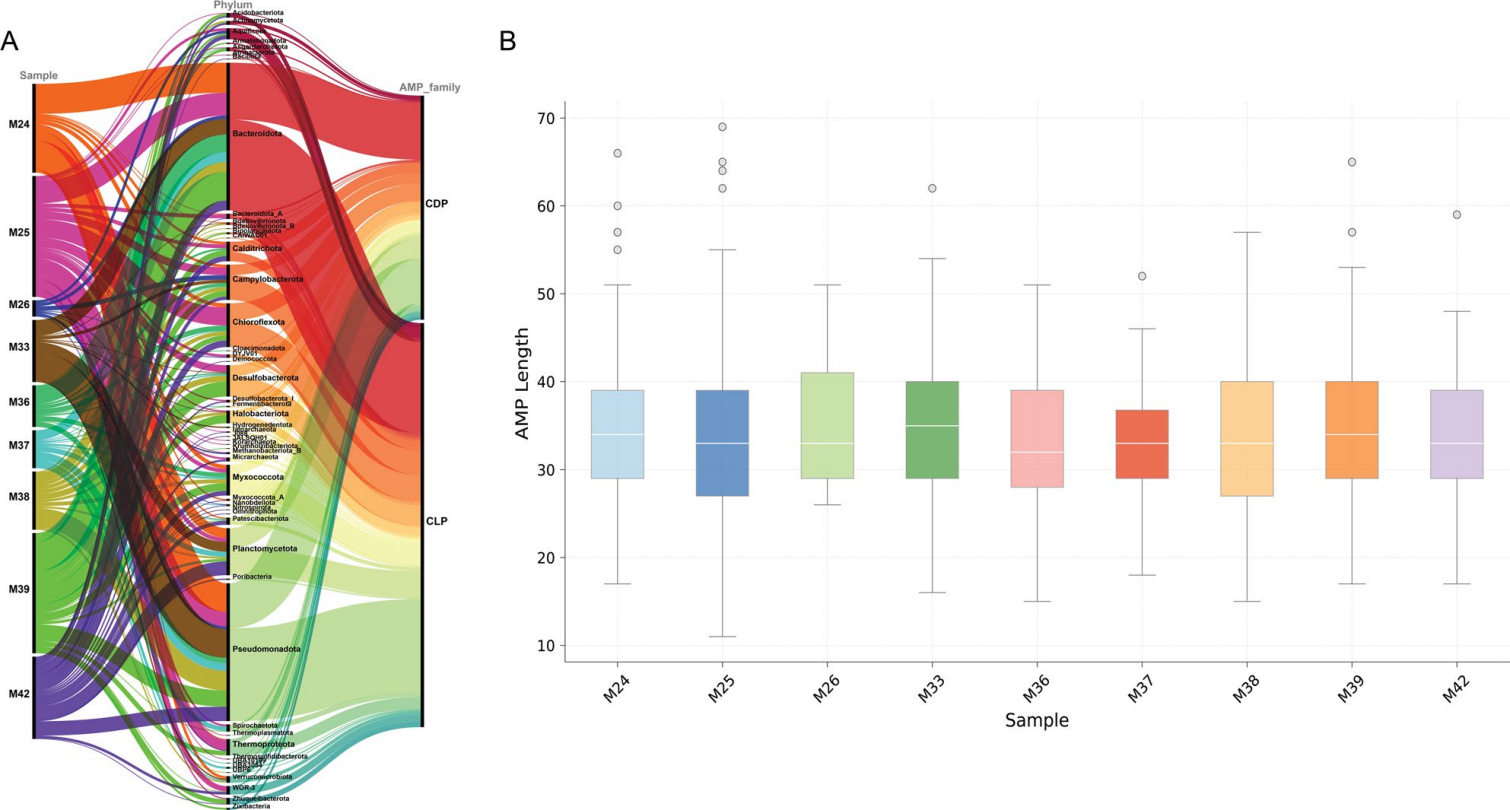
Overview of antimicrobial peptides (AMPs) predicted from hydrothermal vent biofilms. **A** The alluvial diagram illustrates the number of peptide sequences predicted from each biofilm sample, their associated taxonomic affiliations at the phylum level, and the classification of AMPs as either cationic linear peptides (CLPs) or cationic cysteine-containing peptides (CDPs). **B** Average length of AMP sequences predicted from nine biofilm samples

From nine AMOR biofilms, we predicted 961 AMPs using Macrel, among which 873 were unique. The dataset includes 811 singleton AMP sequences, while 62 sequences occur more than once. Overall, AMPs were identified in 51 of the 61 phyla present in the dataset (Fig. 1A).

Of the 961 AMPs, 895 are classified as bacterial, while 66 belong to archaea. The majority of bacterial AMPs were derived from the phyla *Bacteroidota* (226) and *Pseudomonadota* (211). Other notable contributors included *Planctomycetota* (72), *Chloroflexota* (67), and *Campylobacterota* (54). Among the archaea, *Thermoproteota* was the most prevalent, contributing 25 AMP sequences. Archaeal peptides also span a wide range of lineages beyond *Thermoproteota*, including *Halobacteriota* (19), *Micrarchaeota* (6), *Asgardarchaeota* (6), *Nanobdellota* (3), *Methanobacteriota_B* (3), *Iainarchaeota* (2), *Korarchaeota* (1) and *Thermoplasmatota* (1). Detailed information on the AMPs and taxonomic affiliation can be found in Supplementary Table 1.

Sample M25 contained the highest number of AMP sequences (185), while M26 showed the lowest, with only 25 sequences. When classified based on peptide structure, 618 sequences were identified as cationic linear peptides (CLPs), and 343 as cationic peptides with disulfide bonds (CDPs). Additionally, 720 AMPs were predicted to exhibit hemolytic activity, while 241 were classified as non-hemolytic. The comparison between AMPs from AMOR biofilm MAGs and reference AMPs in the APD database revealed no identical matches (Supplementary Table 2).

The peptide sequence “TLLKKPQITQITQIFTRLKLVKIRAIRGFFQ” was the most frequently observed, appearing five times across three MAGs derived from three distinct biofilms: Aegir_M24_B32, Aegir_M25_B215, and Aegir_M36_B21. All three MAGs belong to the bacterial genus JAJRTP01, within the phylum *Chloroflexota*. The longest AMP sequence, consisting of 69 amino acid residues, was found in the bacterial MAG Aegir_M25_B157 (*Desulfobacterota_I*), while the shortest, with 11 residues, was identified in the archaeal MAG Aegir_M25_B19 (*Methanobacteriota_B*). The average length of AMP sequences in the dataset is approximately 33.89 amino acids (Fig. 1B). 68% of the archaeal AMPs were predicted to be hemolytic. These AMPs were not limited to well-known AMP-producing archaeal genera such as *Thermococcus* or *Sulfolobus*, but also included members of underexplored phyla such as *Micrarchaeota*, *Methanobacteriota_B*, *Nanobdellota*, *Korarchaeota*, and *Asgardarchaeota*, taxa that currently have no representatives listed as AMP sources in the APD database.

To assess the contribution of metagenomic sequences not included in MAGs (exMAGs) to AMP prediction, we compared the total number of AMPs, as well as CLP- and CDP-class AMPs, identified from inMAGs versus exMAGs (2169 AMPs; Supplementary Table 7) across the nine biofilms. The Wilcoxon signed-rank test revealed a significantly higher number of AMPs predicted from unbinned contigs compared to inMAGs (*p* = 0.0195), including both CLPs (*p* = 0.0117) and CDPs (*p* = 0.0195) (Supplementary Table 3). The CLP/CDP ratio did not differ significantly between the two sources (*p* = 0.7344). However, unbinned contigscontained ahigher number of AMPscompared to inMAGs. Additionally, the Wilcoxon test indicated a significant difference in the distribution of CLP and CDP classes across microbial metagenomes (*p* = 0.0039) (Supplementary Table 3).Overall, CLPs were more abundant than CDPs .

**Fig. 2:**
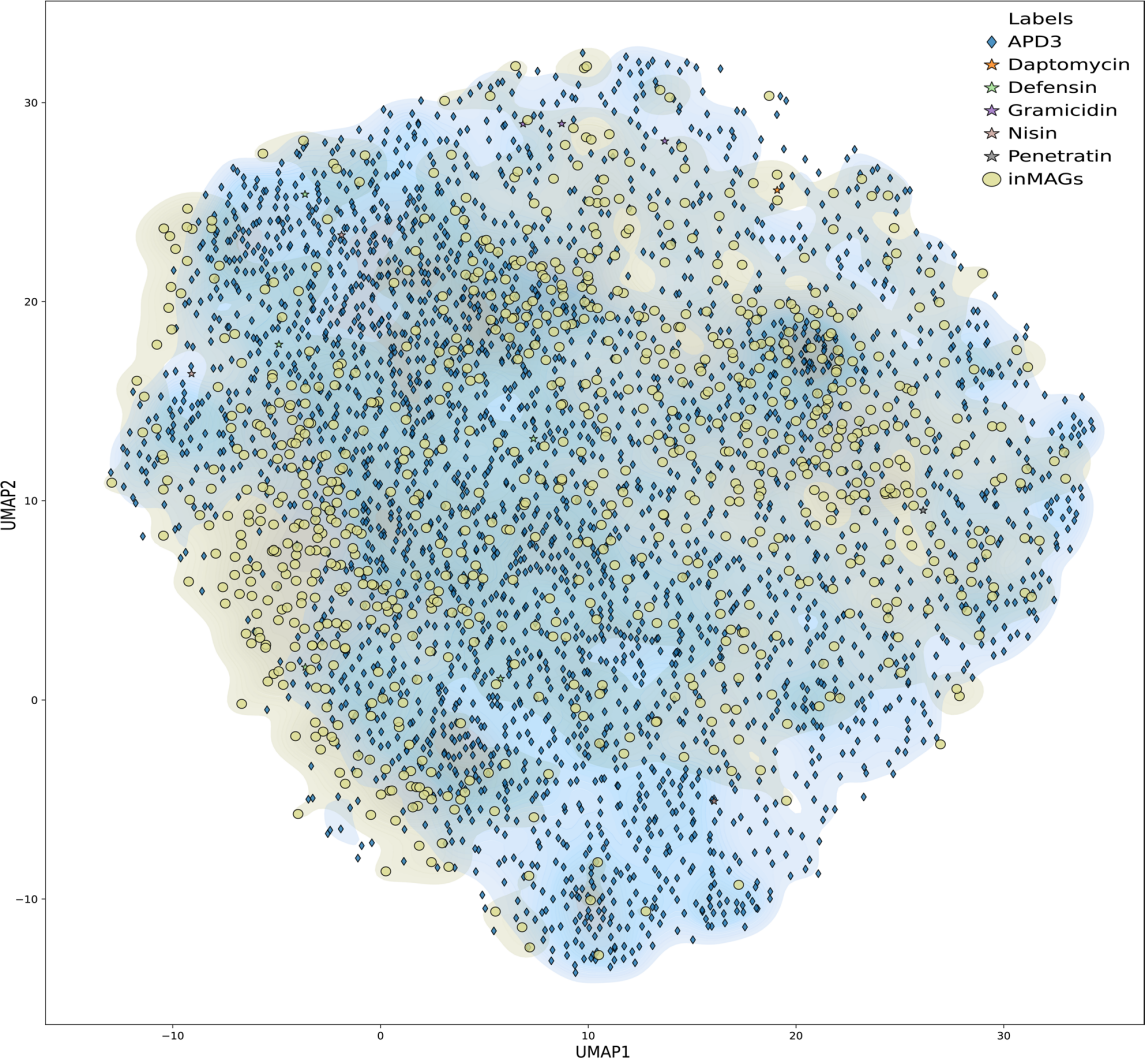
UMAP visualization of nonredundant predicted AMPs from hydrothermal vent biofilms in comparison with AMPs from the APD3 (2024) database. The plot displays the clustering of predicted AMPs alongside known AMPs from the APD3 database. Five FDA-approved reference AMPs, daptomycin, defensin, gramicidin, nisin, and penetratin, are highlighted with star symbols

The UMAP projection revealed the structural diversity of peptides from AMOR biofilms. Peptides from both the APD database and inMAGs formed several distinct and dense clusters (Fig. 2). KDE contours highlighted regions of high peptide concentration, showing multiple zones of overlap between APD and inMAG peptides. Interestingly, some FDA-approved AMPs were positioned close to AMOR-derived peptides. Although limited in number, many of these reference peptides clustered near inMAG sequences, suggesting shared structural features. While inMAG peptides were broadly distributed across the embedding, a substantial portion clustered within or adjacent to APD-defined regions. This spatial co-localization supports the hypothesis that many AMOR peptides may possess antimicrobial properties due to their structural similarity to experimentally validated AMPs. However, further experimental validation is needed to fully verify the connection between structural similarity and proposed antimicrobial activity.

### Diverse taxonomic origins of AMPs revealed through expression profiling

**Fig. 3:**
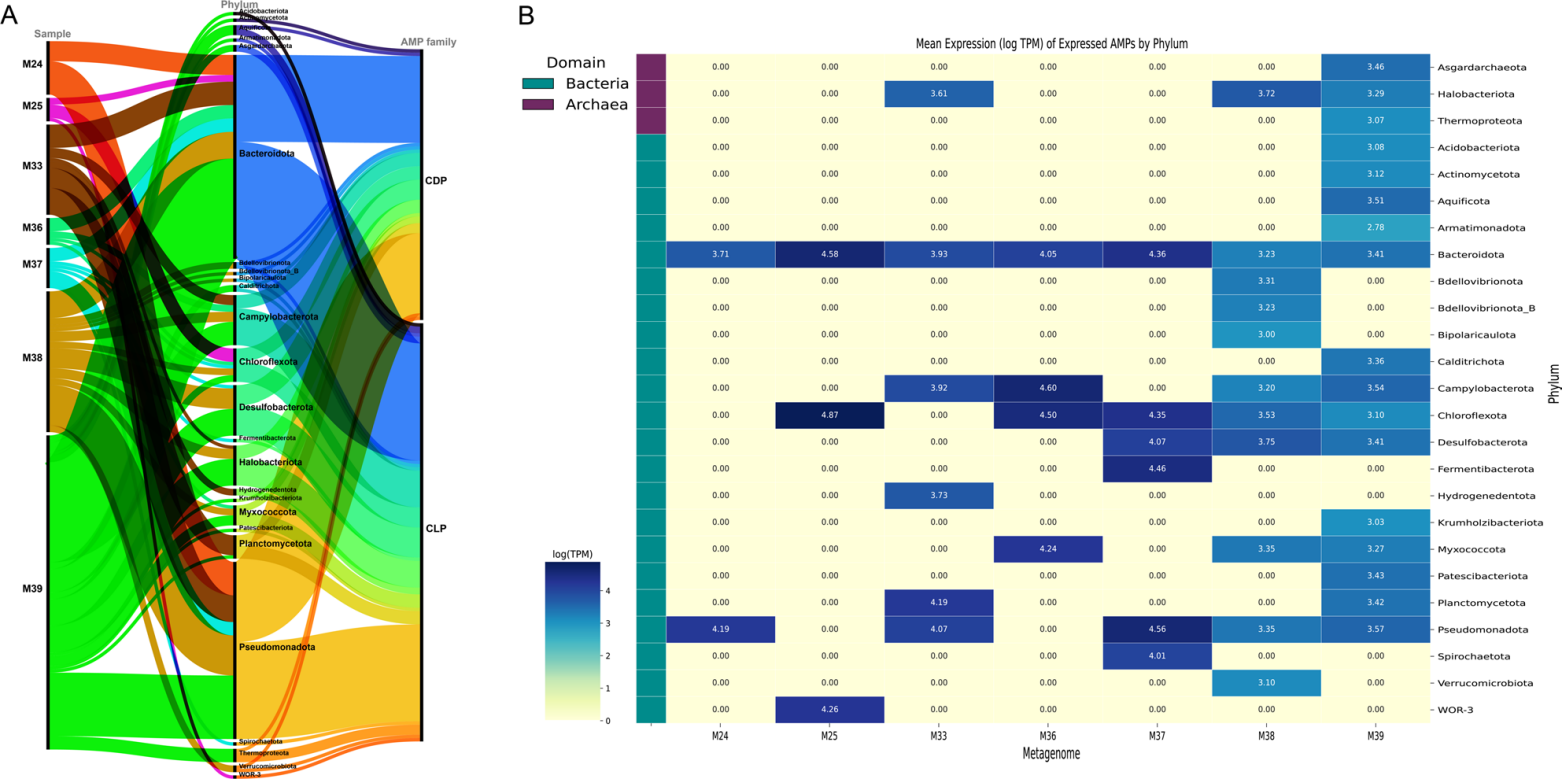
Expression profile of antimicrobial peptides (AMPs) across biofilm-derived MAGs. **A** Alluvial plot showing the distribution and relationships of predicted AMPs based on metatranscriptomic data from seven biofilm samples. The flows illustrate connections between individual samples, microbial taxonomic phyla, and the corresponding AMP families, highlighting number of expressed AMPs, and (**B**) Heatmap showing the mean expression of predicted AMPs across metagenomic samples, grouped by taxonomic phylum

Expression analysis revealed the expression of AMP smORFs originating from 25 distinct phyla, including 22 bacterial and 3 archaeal phyla. Among bacteria, *Bacteroidota* had the highest number of MAGs expressing AMPs (*n* = 43), while within Archaea, *Halobacteriota* was the most transcriptionally active (*n* = 8). Archaeal AMPs were detected exclusively in three biofilms: M33 (1 MAG), M38 (2 MAGs), and notably M39 (10 MAGs). The biofilm M39 also exhibited the highest AMP expression diversity, with peptides originating from 17 different phyla (Fig. 3A). Interestingly, the bacterial phylum *Bacteroidota* was the only group that showed AMP expression in all seven biofilm samples screened (Fig. 3B). In contrast, although 22 AMP sequences from 18 *Pseudomonadota* MAGs were identified in the metagenome of sample M25, none were detected in the corresponding metatranscriptomic data (Fig. 3B). On the other hand, despite representing less than 1% of the microbial community in sample M25, members of the candidate bacterial phylum WOR-3 exhibited high levels of AMP expression (Fig. 3B). All three archaeal phyla with detectable AMP expression were found in sample M39, alongside fourteen bacterial phyla, highlighting this site as a particularly promising location for AMP discovery.

In sample M39, AMP expression was detected in several understudied bacterial phyla, including *Armatimonadota*, *Krumholzibacteriota*, and *Candidatus* Patescibacteria (also known as the Candidate Phyla Radiation- CPR). Despite the reduced genomes of CPR and the absence of many biosynthetic pathway genes, metagenomic data revealed the presence of AMP sequences in their MAGs. Notably, one AMP exhibited moderate expression in MAG Aegir_M39_B87 from sample M39 (log_10_(TPM) = 3.43; Supplementary Table 4). Among the 410 experimentally validated bacterial AMPs listed in the 2024 release of the APD, none originate from these bacterial phyla. The expression of AMPs from typical obligate symbionts or episymbionts, suggests functions beyond classical antimicrobial defense.

**Fig. 4:**
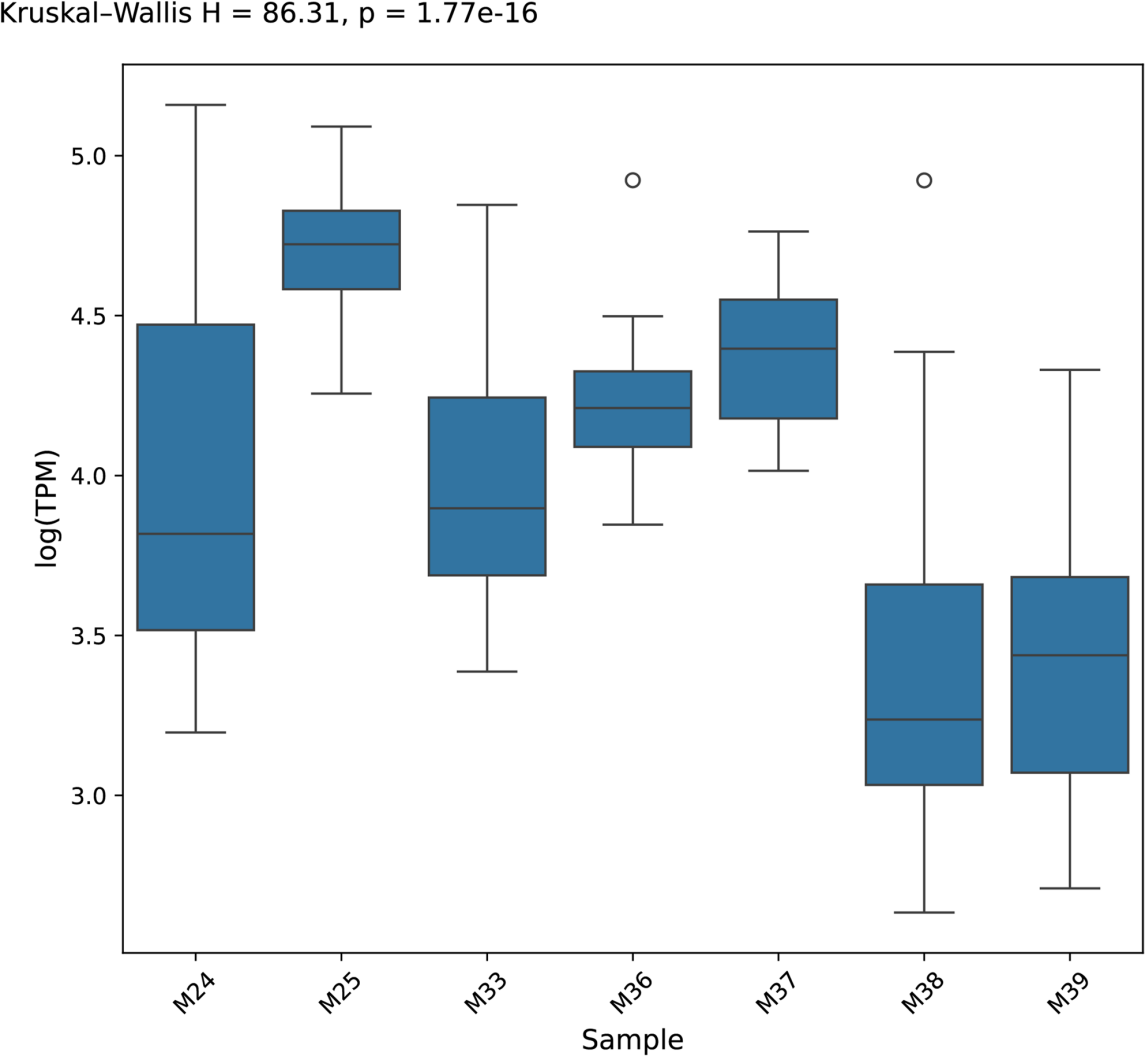
Bar plot of the average expression levels of AMPs across samples, calculated as log10 (TPM). Differences among groups were assessed using the Kruskal–Wallis test, followed by Dunn’s post hoc test with Holm–Bonferroni correction for multiple comparisons. Detailed results of the post hoc analysis, including pairwise comparisons and significance values, are provided in Supplementary Table 6

A Kruskal–Wallis test revealed significant differences in AMP expression levels among the metagenomes (H = 86.31, *p* = 1.77 × 10^− 16^) (Fig. 4). Post hoc Dunn’s test indicated that groups M38 and M39 were significantly different from nearly all other groups (M24, M25, M33, M36, and M37), whereas no significant differences were observed among the other groups (M24-M37) (Supplementary Table 6). Consistent with this, mean TPM values varied substantially between samples: M25 exhibited the highest average expression, driven mainly by three taxa (*Bacteriota*,* Chloroflexota*, and WOR-3), followed by M37 (six taxa: *Bacteroidota*, *Chloroflexota*, *Desulfobacterota*, *Fermentibacterota*, *Pseudomonadota*, and *Spirochaetota*) and M36 (four taxa: *Bacteroidota*, *Campylobacterota*, *Chloroflexota*, and *Myxococcota*) (Figs. 3 and 4). In contrast, M39 displayed the second lowest expression but showed the greatest taxonomic diversity, with up to 17 taxa contributing to AMP transcription (Figs. 3 and 4). These results demonstrate that AMP expression is not uniformly distributed across metagenomes, but instead reflects differences in both expression magnitude and taxonomic breadth.

### Theoretical estimates show promising antimicrobial capacity from deep-sea hydrothermal AMPs

**Fig. 5:**
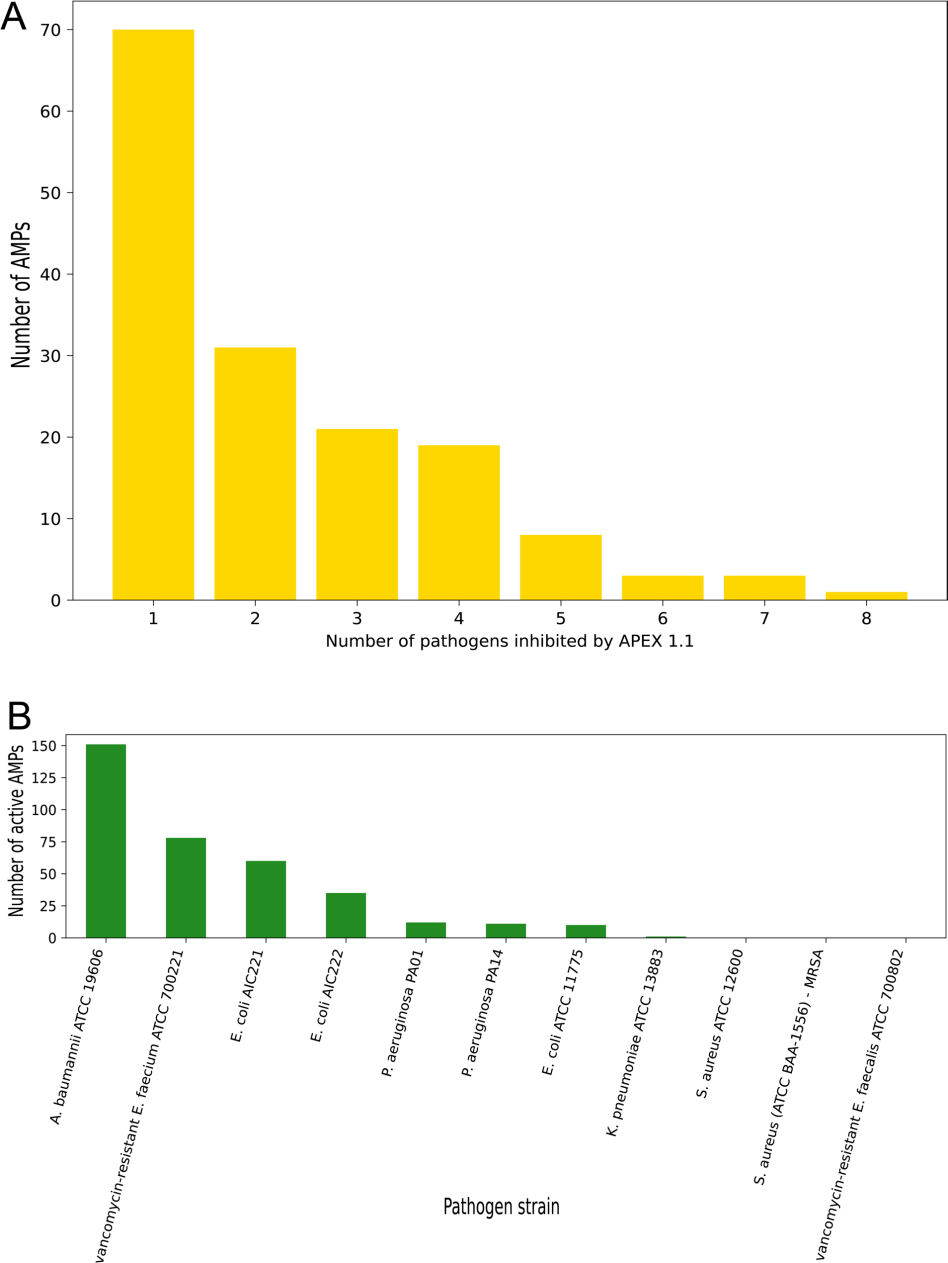
Putative antibacterial activity, the minimal inhibition concentration (MIC), of peptides assessed using the Antibiotic Peptide de-Extinction (APEX) deep learning framework, showing (**A**) the distribution of AMPs by spectrum of pathogen activity, and (**B**) number of AMPs predicted to be active against each pathogen integrated in APEX 1.1

Of the 961 AMPs predicted from the AMOR biofilm metagenomes by Macrel, 933 peptides met the APEX 1.1 input requirement of ≤ 50 amino acids and were subjected to in silico MIC prediction. A total of 156 AMPs were predicted to exhibit antibacterial activity against at least one of the 11 bacterial strains in the APEX 1.1 training panel (defined as MIC < 100 µM) (Supplementary Table 5). Among these, 70 peptides were predicted to inhibit a single pathogen, while 86 peptides displayed high predicted potency against two or more pathogens (Fig. 5A). Especially, one AMP showed predicted activity against eight different bacterial strains, indicating potential broad-spectrum efficacy (Fig. 5A).

*A. baumannii* ATCC 19,606 emerged as the most susceptible strain, with 151 out of 933 peptides (16.2%) predicted to inhibit its growth (Fig. 5B). This was followed by vancomycin-resistant *Enterococcus faecium* ATCC 700,221, for which 78 peptides (8.4%) showed predicted activity. In contrast, no AMPs were predicted to inhibit either of the two *Staphylococcus aureus* strains (ATCC 12600 and MRSA ATCC BAA-1556) or vancomycin-resistant *Enterococcus faecalis* ATCC 700,802 (Fig. 5B), suggesting high resistance of these strains to the AMOR-derived peptides in silico.

### Antimicrobial activity of deep-sea hydrothermal AMPs

Four AMPs were selected for experimental validation and tested in vitro against *E. coli* and *S. aureus* using broth microdilution assays. Of these, two peptides exhibited antibacterial activity. AMP OLKFNNDA_52_10 showedmoderate inhibitory activity against *S. aureus* andweak activity against *E. coli*, while MENBPKOE_145_15 demonstrated weak inhibition of *S. aureus* (Table 1).

**Table 1:** Minimum inhibitory concentration (MIC) and cytotoxicity results of four AMPs selected from the AMOR biofilm dataset. Peptides were randomly chosen from the 961 AMP candidates based on selection criteria described in the Methods. MIC assays were performed against *Escherichia coli* K-12 MG1655 and *Staphylococcus aureus* ATCC 29,213. Effects on growth in mammalian cells were measured in HEK293 cell line. Two independent biological replicates were performed

MAG ID	AMP_ID	MIC E. coli [µg/ml]	MIC S. aureus [µg/ml]	IC50 HEK [µM]
Aegir_M38_B6	LPEMMCJN_72_9	> 128	> 128	> 10
Aegir_M25_B26	MENBPKOE_145_15	> 128	128	6–8
Aegir_M24_B77	OLKFNNDA_52_10	128	64	> 10
Aegir_M24_B17	JECBHHKC_163_2	> 128	> 128	> 10

The remaining two peptides (JECBHHKC_163_2 and LPEMMCJN_72_9) showed no detectable inhibition against either strain (Table 1). Among the four peptides tested, OLKFNNDA_52_10 was the only one predicted by APEX 1.1 to possess antimicrobial activity against multiple pathogens, with MIC values < 100 µM for *A. baumannii* ATCC 19,606, *E. coli* AIC221, and vancomycin-resistant *E. faecium* ATCC 700,221 (Supplementary Table 5). Although APEX predicted none of the peptides to be active against *S. aureus*, OLKFNNDA_52_10 exhibited moderate in vitro inhibition of the strain, highlighting a discrepancy between model prediction and biological outcome. Similarly, MENBPKOE_145_15, which was not predicted to be active against any APEX pathogens, showed weak activity against *S. aureus*. In contrast, JECBHHKC_163_2, which was predicted to inhibit *A. baumannii* ATCC 19,606, did not display any activity against the tested strains. The viability assay showed that three of the four tested AMPs were well tolerated by the human HEK293 cell line, while MENBPKOE_145_15 had growth inhibiting effects (Table 1).

## Discussion

The global rise of antimicrobial resistance has intensified the search for novel antimicrobial agents, shifting attention from well-characterized producers such as *Actinomycetota* to underexplored microbial lineages [13, 18, 29]. Extremophiles have gained particular interest because their proteins are adapted to harsh conditions, making them more stable and potentially more effective across a wide range of environmental conditions [15, 18, 30]. Torres et al. [15] proposed that thermophilic adaptation may be linked to increased production of bioactive peptides. Our findings support this hypothesis and demonstrate that biofilms from the AMOR represent a rich and previously untapped reservoir of AMPs.

Our multi-omics analyses revealed that AMPs in AMOR biofilms are encoded by a phylogenetically diverse array of microorganisms, including several phyla with no prior AMP entries in public databases. While bacterial groups such as *Pseudomonadota* and *Bacteroidota* were dominant contributors, archaeal taxa also played a significant role, with 66 peptides attributed to this domain. Within the archaea, *Thermoproteota* harbored the highest number of AMP-encoding MAG, consistent with previous reports of archaeal AMPs such as sulfolobicins and halocins [31]. The presence of AMPs in underexplored phyla such as *Thermoplasmatota*, *Nanobdellota*, and *Asgardarchaeota* was recently reported [15]. In this study, we confirmed their presence using metagenomic data, and in the case of *Asgardarchaeota*, with transcriptomic support for AMP expression. The absence of these lineages in AMP databases likely reflects the limited availability of reference genomes rather than a true lack of biosynthetic potential [15]. These findings underscore the need for continued exploration of extreme environments to better understand the diversity and function of archaeal AMPs.

Beyond taxonomic novelty, the AMPs identified in AMOR biofilms exhibited high sequence diversity. None of the predicted peptides matched known entries in the APD database, although clustering analyses revealed proximity to characterized AMPs, suggesting shared structural or functional features [15, 32]. Given that even single amino acid substitutions can significantly alter AMP activity [32, 33], the observed sequence variation represents a valuable resource for the discovery of novel antimicrobial scaffolds. AMP production was also observed among closely related species within the biofilms, raising the possibility of cooperative antimicrobial strategies. Such synergistic activity may be advantageous in extreme environments, where microbial communities must balance competition and cooperation to survive [15]. The comparable CLP/CDP ratio between unbinned contigs and inMAGs suggests that, despite differences in total AMP abundance, the structural composition of these peptide classes is conserved across sources. Furthermore, the higher abundance of CLPs relative to CDPs may indicate distinct functional roles of these peptide classes within AMOR biofilms.

Environmental context also appeared to influence AMP expression. Sample M39 emerged as a particularly promising site, with transcriptional activity detected across 17 phyla, including rare and uncultured archaeal taxa such as *Asgardarchaeota* and CPR [28, 34]. The ultra-small CPR bacteria are often shown to live in close association with host microbes [35–37]. The ecological roles of AMPs in CPR bacteria remain poorly understood, but several hypotheses can be proposed. AMPs may modulate host physiology or immunity [38, 39], helping CPR bacteria maintain stable symbiotic relationships. Alternatively, they may suppress competing microbes within the biofilm, indirectly benefiting the host and stabilizing the niche CPR bacteria depend on. Some CPR-derived AMPs may also serve protective functions, helping cells cope with environmental stressors such as oxidative damage [40] or metal toxicity, conditions common in hydrothermal systems. Given that certain AMPs possess metal-binding properties, they may also contribute to metal homeostasis or detoxification, in addition to boost antibacterial activity [41]. Furthermore, CPR AMPs could influence the metabolic activity of neighboring microbes, ensuring the availability of essential metabolites, or inhibit specific enzymes and transporters in competitors. Some AMPs may even act as anti-quorum sensing molecules [42], shaping microbial behavior and biofilm architecture in ways that promote CPR survival. Finally, these peptides may defend CPR cells or their hosts against protozoan grazers, reinforcing the symbiotic bond. These hypotheses warrant further investigation, particularly through functional assays and co-culture experiments.

Archaea from extreme environments are known to produce highly stable antimicrobial compounds, archaeasins, that remain active under complex biological conditions [15, 43]. These properties make them attractive candidates for pharmaceutical development, where stability and efficacy are critical [18]. Our detection of AMP transcripts in uncultured lineages such as UBA1924 (*Planctomycetota*) and JAGGXT01 (*Asgardarchaeota*) highlights the challenges of studying AMP biosynthesis in uncultivated organisms. However, by integrating multi-omics approaches with synthetic biology and computational modeling, it is possible to characterize and optimize these peptides for downstream applications. Collectively, our results expand the known diversity of archaeal and CPR-derived AMPs and demonstrate that AMOR biofilms harbor a unique and largely untapped reservoir of antimicrobial potential. These findings emphasize the importance of exploring extreme environments and underrepresented microbial lineages in the ongoing search for next-generation antimicrobial agents.

This study also highlights the importance of integrating in silico prediction tools with in vitro validation to expedite AMP discovery. Using APEX 1.1, we predicted that 16.7% of AMPs identified from the AMOR biofilm metagenomes may possess antimicrobial activity, including candidates with broad-spectrum potential. Particularly, *A. baumannii*, a World Health Organization (WHO) priority pathogen [44, 45], emerged as the most susceptible target, with 151 peptides predicted to exhibit MIC values below 100 µM. Experimental validation supported some of the computational predictions. AMP OLKFNNDA_52_10, predicted to be active against multiple pathogens, showed moderate in vitro activity against *S. aureus* and weak activity against *E. coli*. Interestingly, this peptide was not predicted to inhibit *S. aureus*, suggesting that current models may fail to capture all the structural factors that influence antimicrobial function. Given its demonstrated antibacterial activity and low cytotoxicity toward the tested human cell line, OLKFNNDA_52_10 represents a promising candidate for further development as a potential antimicrobialdrug. The results highlight OLKFNNDA_52_10 as a particularly promising AMP candidate, as it combines antibacterial activity against the tested pathogens with good tolerability in human cells.

In contrast, the weak activity observed for MENBPKOE_145_15, despite no predicted efficacy, highlights the potential for false negatives and underscores the need for continued refinement of machine learning models. These findings further illustrate the complexity of AMP screening and prediction. Recent studies have also shown that synergistic interactions among AMPs within microbial communities can amplify antimicrobial effects [15]. Such community-level interactions remain beyond the scope of current bioinformatic prediction tools and therefore require in vitro validation, reinforcing the indispensable role of experimental assays in AMP discovery.

Overall, our findings demonstrate that AMOR hydrothermal vent biofilms are a rich and largely untapped source of diverse AMPs with antimicrobial potential. The mismatch between the predictions and experimental results highlights a crucial point: while machine learning models are valuable for narrowing the search space, they often fall short in capturing the full complexity of AMP activity. This reinforces the essential role of in vitro testing in AMP research, not only to validate predictions, but to reveal bioactive peptides that models may miss. By integrating transcriptomic evidence, computational screening, and experimental validation, we can establish a more reliable and comprehensive framework for discovering novel antimicrobials from underexplored microbiomes. We emphasize that only 4 peptides in the current study were subjected to experimental validation, hence additional peptides need to be validated to assess the full antimicrobial potential of these biofilms, including extending the validation to mechanistic assays to assess mode of action. Furthermore, to extend the discovery workflow beyond proof-of-concept that AMOR biofilms have a great AMP potential, a combination of AI-assisted tools may be employed.

## Supplementary Information

Supplementary Material 1: Table S1. Predicted antimicrobial peptides from AMOR hydrothermal vent biofilms. Peptide prediction was performed using Macrel v1.2.0, and the results include AMP class assignments, hemolytic probability, and AMP probability scores. Taxonomic classification of all MAGs was conducted using GTDB-Tk v2.5.2 with database release R226.

Supplementary Material 2: Table S2. CD-HIT clustering of predicted AMPs from inMAGs and reference AMP sequences from the APD database (−c 1, −n 5). No inMAG sequences were identical to experimentally validated AMPs in the 2024 APD release.

Supplementary Material 3: Table S3. Results of the Wilcoxon signed-rank test evaluating differences in peptide class abundance between CLP and CDP in the inMAGs dataset.

Supplementary Material 4: Table S4. Expression profiles of small open reading frames (smORFs) containing predicted antimicrobial peptide sequences from inMAGs.

Supplementary Material 5: Table S5. In silico minimum inhibitory concentration (MIC) predictions for inMAG-derived antimicrobial peptides generated using APEX 1.1. Predictions were obtained from a model trained on antimicrobial activities against 11 human pathogenic strains.

Supplementary Material 6: Table S6. Kruskal–Wallis test showing differences in AMP expression levels among the Arctic deep-sea hydrothermal metagenomes. Post hoc Dunn’s tests with Holm-Bonferroni correction were performed to identify significant pairwise differences.

Supplementary Material 7: Table S7. Predicted antimicrobial peptides identified from contigs not included in MAGs (exMAGs). Four AMP family types are represented in this dataset: CLP (cationic linear peptides), CDP (cationic peptides with disulfide bonds), ALP (anionic linear peptides), and ADP (anionic peptides with disulfide bonds). A total of 2,169 peptides were predicted from these contigs.

## Data Availability

All data generated and analysed during this study are included in this published article (and its supplementary information files). The datasets analysed in the current study was recently published [19] and are available via BioProjects; PRJNA1301805, PRJNA881934, PRJNA1301797, , PRJNA1301809, PRJNA1301812, PRJNA1301820, PRJNA1301824 and , with MAG sequence data and raw sequencing data deposited at NCBI. The modified main.py script for longread-abundance, along with all source code and data used for statistical analyses, is available on GitHub ( https://github.com/trongthucnguyen/DeepSeaQuence_biofilms ).

## References

[1] Samgina TYu, Vorontsov EA, Gorshkov VA, Hakalehto E, Hanninen O, Zubarev RA, et al. Composition and Antimicrobial Activity of the Skin Peptidome of Russian Brown Frog *Rana temporaria*. J Proteome Res Am Chem Soc (ACS). 2012;11:6213–22. 10.1021/pr300890m. 10.1021/pr300890m 23121565

[2] Chettri D, Rani A, Sharma B, Selvaraj M, Assiri MA, Verma AK. Antimicrobial peptides: Source, application and recent developments. Process Biochem Elsevier BV. 2024;145:288–301. 10.1016/j.procbio.2024.07.002.

[3] Oliveira Júnior NG, Souza CM, Buccini DF, Cardoso MH, Franco OL. Antimicrobial peptides: structure, functions and translational applications. Nat Rev Microbiol Springer Sci Bus Media LLC. 2025. 10.1038/s41579-025-01200-y. [cited 2025 Aug 21]. 10.1038/s41579-025-01200-y 40646173

[4] Yang R, Ma X, Peng F, Wen J, Allahou LW, Williams GR, et al. Advances in antimicrobial peptides: From mechanistic insights to chemical modifications. Biotechnol Adv Elsevier BV. 2025;81:108570. 10.1016/j.biotechadv.2025.108570. 10.1016/j.biotechadv.2025.108570 40154761

[5] Santos-Júnior CD, Torres MDT, Duan Y, Rodríguez Del Río Á, Schmidt TSB, Chong H et al. Discovery of antimicrobial peptides in the global microbiome with machine learning. Cell. 2024;187:3761–3778.e16. 10.1016/j.cell.2024.05.013. 10.1016/j.cell.2024.05.013 PMC11666328 38843834

[6] Ayikpoe RS, Shi C, Battiste AJ, Eslami SM, Ramesh S, Simon MA, et al. A scalable platform to discover antimicrobials of ribosomal origin. Nat Commun. 2022;13:6135. 10.1038/s41467-022-33890-w. 36253467 10.1038/s41467-022-33890-w PMC9576775

[7] Agrawal S, Acharya D, Adholeya A, Barrow CJ, Deshmukh SK. Nonribosomal Peptides from Marine Microbes and Their Antimicrobial and Anticancer Potential. Front Pharmacol Front Media SA. 2017. 10.3389/fphar.2017.00828. [cited 2025 Aug 20];8. 10.3389/fphar.2017.00828 PMC5702503 29209209

[8] Bogdanov IV, Fateeva SI, Voropaev AD, Ovchinnikova TV, Finkina EI. Immunomodulatory Effects of the Pea Defensin Psd1 in the Caco-2/Immune Cells Co-Culture upon Candida albicans Infection. Int J Mol Sci MDPI AG. 2023;24:7712. 10.3390/ijms24097712. 10.3390/ijms24097712 PMC10178127 37175419

[9] Ramos R, Moreira S, Rodrigues A, Gama M, Domingues L. Recombinant expression and purification of the antimicrobial peptide magainin-2. Biotechnol Prog Wiley. 2013;29:17–22. 10.1002/btpr.1650. 10.1002/btpr.1650 23125137

[10] Tornesello AL, Borrelli A, Buonaguro L, Buonaguro FM, Tornesello ML. Antimicrobial Peptides as Anticancer Agents: Functional Properties and Biological Activities. Molecules MDPI AG. 2020;25:2850. 10.3390/molecules25122850. 10.3390/molecules25122850 PMC7356147 32575664

[11] Le VH, Inai M, Williams RM, Kan T, Ecteinascidins. A review of the chemistry, biology and clinical utility of potent tetrahydroisoquinoline antitumor antibiotics. Nat Prod Rep Royal Soc Chem (RSC). 2015;32:328–47. 10.1039/c4np00051j. 10.1039/c4np00051j PMC4806878 25273374

[12] Spohn R, Daruka L, Lázár V, Martins A, Vidovics F, Grézal G, et al. Integrated evolutionary analysis reveals antimicrobial peptides with limited resistance. Nat Commun Springer Sci Bus Media LLC. 2019. 10.1038/s41467-019-12364-6. [cited 2025 Aug 21];10. 10.1038/s41467-019-12364-6 PMC6778101 31586049

[13] World Health Organization. Global action plan on antimicrobial resistance . Geneva: World Health Organization. 2015 [cited 2024 May 28]. https://iris.who.int/handle/10665/193736. Accessed 28 May 2024.

[14] Dadgostar P. Antimicrobial Resistance: Implications and Costs. Infect Drug Resist. 2019;12:3903–10. 10.2147/IDR.S234610. 31908502 10.2147/IDR.S234610 PMC6929930

[15] Torres MDT, Wan F, De La Fuente-Nunez C. Deep learning reveals antibiotics in the archaeal proteome. Nat Microbiol Springer Sci Bus Media LLC. 2025. 10.1038/s41564-025-02061-0. [cited 2025 Aug 22]. 10.1038/s41564-025-02061-0 PMC12408343 40796684

[16] Jangra M, Travin DY, Aleksandrova EV, Kaur M, Darwish L, Koteva K, et al. A broad-spectrum lasso peptide antibiotic targeting the bacterial ribosome. Nat Springer Sci Bus Media LLC. 2025;640:1022–30. 10.1038/s41586-025-08723-7. 10.1038/s41586-025-08723-7 PMC12497486 40140562

[17] Mallapaty S. New antibiotic that kills drug-resistant bacteria discovered in technician’s garden. Nat Springer Sci Bus Media LLC. 2025;640:19–20. 10.1038/d41586-025-00945-z. 10.1038/d41586-025-00945-z 40140515

[18] Quinn GA, Dyson PJ. Going to extremes: progress in exploring new environments for novel antibiotics. Npj Antimicrob Resist . Springer Science and Business, Media LLC. 2024;2. 10.1038/s44259-024-00025-8. Cited 2025 Aug 22. 10.1038/s44259-024-00025-8 PMC11721673 39843508

[19] Nguyen TT, Steen IH, Stokke R. Remarkable biosynthetic capacity of Arctic hydrothermal biofilms . In Review; 2025. 10.21203/rs.3.rs-7619139/v1. Cited 2025 Nov 12.

[20] Zhao X, Wang X, Shukla R, Kumar R, Weingarth M, Breukink E, et al. Brevibacillin 2V, a Novel Antimicrobial Lipopeptide With an Exceptionally Low Hemolytic Activity. Front Microbiol Front Media SA. 2021. 10.3389/fmicb.2021.693725. [cited 2025 Aug 20];12. 10.3389/fmicb.2021.693725 PMC8245773 34220785

[21] Santos-Júnior CD, Pan S, Zhao X-M, Coelho LP. Macrel: antimicrobial peptide screening in genomes and metagenomes. PeerJ. 2020;8:e10555. 10.7717/peerj.10555. 33384902 10.7717/peerj.10555 PMC7751412

[22] Wang G, Li X, Wang Z. APD3: the antimicrobial peptide database as a tool for research and education. Nucleic Acids Res. 2016;44:D1087–93. 10.1093/nar/gkv1278. 26602694 10.1093/nar/gkv1278 PMC4702905

[23] Li W, Godzik A. Cd-hit: a fast program for clustering and comparing large sets of protein or nucleotide sequences. Bioinformatics. 2006;22:1658–9. 10.1093/bioinformatics/btl158. 16731699 10.1093/bioinformatics/btl158

[24] McInnes L, Healy J, Melville J. UMAP: Uniform Manifold Approximation and Projection for Dimension Reduction . arXiv; 2018. 10.48550/ARXIV.1802.03426. Cited 2025 Apr 22.

[25] Wan F, Torres MDT, Peng J, de la Fuente-Nunez C. Deep-learning-enabled antibiotic discovery through molecular de-extinction. Nat Biomed Eng Nat Publishing Group. 2024;8:854–71. 10.1038/s41551-024-01201-x. 10.1038/s41551-024-01201-x PMC11310081 38862735

[26] Miller WR, Arias CA. ESKAPE pathogens: antimicrobial resistance, epidemiology, clinical impact and therapeutics. Nat Rev Microbiol. 2024;22:598–616. 10.1038/s41579-024-01054-w. 38831030 10.1038/s41579-024-01054-w PMC13147291

[27] Cockerill FR. Clinical and Laboratory Standards Institute, editors. Methods for dilution antimicrobial susceptibility tests for bacteria that grow aerobically: approved standard - ninth edition. Wayne, Pa: CLSI; 2012.

[28] Srinivas P, Peterson SB, Gallagher LA, Wang Y, Mougous JD. Beyond genomics in Patescibacteria: A trove of unexplored biology packed into ultrasmall bacteria. Proc Natl Acad Sci. 2024;121:e2419369121. 10.1073/pnas.2419369121. 39665754 10.1073/pnas.2419369121 PMC11665869

[29] Murray CJL, Ikuta KS, Sharara F, Swetschinski L, Robles Aguilar G, Gray A, et al. Global burden of bacterial antimicrobial resistance in 2019: a systematic analysis. Lancet. 2022;399:629–55. 10.1016/S0140-6736(21)02724-0. 35065702 10.1016/S0140-6736(21)02724-0 PMC8841637

[30] Sayed AM, Hassan MHA, Alhadrami HA, Hassan HM, Goodfellow M, Rateb ME. Extreme environments: microbiology leading to specialized metabolites. J Appl Microbiol. 2020;128:630–57. 10.1111/jam.14386. 31310419 10.1111/jam.14386

[31] O’Connor E, Shand R. Halocins and sulfolobicins: The emerging story of archaeal protein and peptide antibiotics. J Ind Microbiol Biotechnol. Volume 28. Oxford University Press (OUP); 2002;23–31. 10.1038/sj/jim/7000190. 10.1038/sj/jim/7000190 11938468

[32] Li T, Ren X, Luo X, Wang Z, Li Z, Luo X, et al. A Foundation Model Identifies Broad-Spectrum Antimicrobial Peptides against Drug-Resistant Bacterial Infection. Nat Commun. 2024;15:7538. 10.1038/s41467-024-51933-2. 39214978 10.1038/s41467-024-51933-2 PMC11364768

[33] Lei M, Jayaraman A, Van Deventer JA, Lee K. Engineering Selectively Targeting Antimicrobial Peptides. Annu Rev Biomed Eng Annual Reviews. 2021;23:339–57. 10.1146/annurev-bioeng-010220-095711. 10.1146/annurev-bioeng-010220-095711 PMC9017812 33852346

[34] Harris JK, Kelley ST, Pace NR. New Perspective on Uncultured Bacterial Phylogenetic Division OP11. Appl Environ Microbiol. 2004;70:845–9. 10.1128/AEM.70.2.845-849.2004. 14766563 10.1128/AEM.70.2.845-849.2004 PMC348892

[35] He Y, Zhuo S, Li M, Pan J, Jiang Y, Hu Y, et al. Candidate Phyla Radiation (CPR) bacteria from hyperalkaline ecosystems provide novel insight into their symbiotic lifestyle and ecological implications. Microbiome. 2025;13:94. 10.1186/s40168-025-02077-y. 40189564 10.1186/s40168-025-02077-y PMC11974145

[36] Kuroda K, Yamamoto K, Nakai R, Hirakata Y, Kubota K, Nobu MK et al. SJ <>Giovannoni editor 2022 Symbiosis between Candidatus Patescibacteria and Archaea Discovered in Wastewater-Treating Bioreactors. mBio 13 e01711–22 10.1128/mbio.01711-22. 36043790 10.1128/mbio.01711-22 PMC9600506

[37] Wang Y, Gallagher LA, Andrade PA, Liu A, Humphreys IR, Turkarslan S, et al. Genetic manipulation of Patescibacteria provides mechanistic insights into microbial dark matter and the epibiotic lifestyle. Cell. 2023;186:4803–e481713. 10.1016/j.cell.2023.08.017. 37683634 10.1016/j.cell.2023.08.017 PMC10633639

[38] Duarte-Mata DI, Salinas-Carmona MC. Antimicrobial peptides´ immune modulation role in intracellular bacterial infection. Front Immunol. 2023;14:1119574. 10.3389/fimmu.2023.1119574. 37056758 10.3389/fimmu.2023.1119574 PMC10086130

[39] Le Bloa S, Boidin-Wichlacz C, Cueff-Gauchard V, Rosa RD, Cuvillier-Hot V, Durand L, et al. Antimicrobial Peptides and Ectosymbiotic Relationships: Involvement of a Novel Type IIa Crustin in the Life Cycle of a Deep-Sea Vent Shrimp. Front Immunol. 2020;11:1511. 10.3389/fimmu.2020.01511. 32765521 10.3389/fimmu.2020.01511 PMC7381244

[40] Choi H, Yang Z, Weisshaar JC. Oxidative stress induced in E. coli by the human antimicrobial peptide LL-37. Yeaman MR. editor PLOS Pathog. 2017;13:e1006481. 10.1371/journal.ppat.1006481. 10.1371/journal.ppat.1006481 PMC5509375 28665988

[41] Wang C, Wei X, Zhong L, Chan C-L, Li H, Sun H. Metal-Based Approaches for the Fight against Antimicrobial Resistance: Mechanisms, Opportunities, and Challenges. J Am Chem Soc Am Chem Soc. 2025;147:12361–80. 10.1021/jacs.4c16035. 10.1021/jacs.4c16035 PMC12007004 40063057

[42] Castillo-Juárez I, Blancas-Luciano BE, García-Contreras R, Fernández-Presas AM. Antimicrobial peptides properties beyond growth inhibition and bacterial killing. PeerJ. 2022;10:e12667. 10.7717/peerj.12667. 35116194 10.7717/peerj.12667 PMC8785659

[43] Riley MA, Chavan MA, editors. Bacteriocins: Ecology and Evolution. Berlin, Heidelberg: Springer- Berlin Heidelberg; 2007. 10.1007/978-3-540-36604-1.

[44] WHO Bacterial Priority Pathogens List. 2024: Bacterial Pathogens of Public Health Importance, to Guide Research, Development, and Strategies to Prevent and Control Antimicrobial Resistance. 1st ed. Geneva: World Health Organization; 2024.

[45] Foglia F, Ambrosino A, Bashir S, Finamore E, Zannella C, Donnarumma G, et al. Prevalence of Acinetobacter baumannii Multidrug Resistance in University Hospital Environment. Antibiotics. 2025;14:490. 10.3390/antibiotics14050490. 40426554 10.3390/antibiotics14050490 PMC12108267

